# Generation of Direct-, Retrograde-, and Source-Wave Gaits in Multi-Legged Locomotion in a Decentralized Manner via Embodied Sensorimotor Interaction

**DOI:** 10.3389/fncir.2021.706064

**Published:** 2021-09-06

**Authors:** Yuichi Ambe, Shinya Aoi, Kazuo Tsuchiya, Fumitoshi Matsuno

**Affiliations:** ^1^Tough Cyberphysical AI Research Center, Tohoku University, Sendai, Japan; ^2^Department of Aeronautics and Astronautics, Kyoto University, Kyoto, Japan; ^3^Department of Mechanical Engineering and Science, Kyoto University, Kyoto, Japan

**Keywords:** interlimb coordination, multi-legged locomotion, millipede, metachronal waves, local sensory feedback, embodied sensorimotor interaction

## Abstract

Multi-legged animals show several types of ipsilateral interlimb coordination. Millipedes use a direct-wave gait, in which the swing leg movements propagate from posterior to anterior. In contrast, centipedes use a retrograde-wave gait, in which the swing leg movements propagate from anterior to posterior. Interestingly, when millipedes walk in a specific way, both direct and retrograde waves of the swing leg movements appear with the waves' source, which we call the source-wave gait. However, the gait generation mechanism is still unclear because of the complex nature of the interaction between neural control and dynamic body systems. The present study used a simple model to understand the mechanism better, primarily how local sensory feedback affects multi-legged locomotion. The model comprises a multi-legged body and its locomotion control system using biologically inspired oscillators with local sensory feedback, phase resetting. Each oscillator controls each leg independently. Our simulation produced the above three types of animal gaits. These gaits are not predesigned but emerge through the interaction between the neural control and dynamic body systems through sensory feedback (embodied sensorimotor interaction) in a decentralized manner. The analytical description of these gaits' solution and stability clarifies the embodied sensorimotor interaction's functional roles in the interlimb coordination.

## 1. Introduction

Multi-legged animals, even those with a large number of legs, use several types of ipsilateral interlimb coordination according to the species and situations. Centipedes use a retrograde-wave gait in which the swing leg movements propagate from the anterior to posterior (Full, 1997; Kuroda et al., 2014). In contrast, millipedes generally use a direct-wave gait, in which the swing leg movements propagate from the posterior to anterior (Full, 1997; Kuroda et al., 2014). More interestingly, it is reported that when millipedes walk with the body axis bent like a U shape, both direct and retrograde waves of the swing leg movements appear at the source of the waves (Tamura et al., 2016), which we call the source-wave gait. However, it remains unclear what mechanisms generate these different types of interlimb coordination in multi-legged locomotion.

Regarding the locomotion of insects and mammals that use a direct-wave gait, where the swing leg movements propagate from the posterior to anterior, physiological studies have suggested that central pattern generators (CPGs) and sensory feedback play important roles in the interlimb coordination (Delcomyn, 2004; Büschges et al., 2008; Ijspeert, 2008). The CPGs generate rhythmic outputs, which are modulated by sensory feedback. The sensory feedback is critical especially during slow walking, as seen in stick insects (Delcomyn, 2004; Büschges et al., 2008).

However, the mechanism of the interlimb coordination has not been fully understood only from physiological studies because the locomotion is generated through complex interactions between motor control and body dynamics through sensory feedback (embodied sensorimotor interaction). Thus, many mathematical models and robots have been developed to clarify the functional roles of these interactions in the interlimb coordination (Steingrube et al., 2010; Owaki et al., 2012; Aoi et al., 2013; Schilling et al., 2013; Ambe et al., 2015).

Recently, mathematical models and robots for multi-legged locomotion have reproduced many aspects of the gaits of multi-legged animals by focusing on the embodied sensorimotor interaction. Tamura et al. (2016) showed that sensory feedback of load information generates a millipede-like gait. Yasui et al. (2017) and Kano et al. (2017) proposed distributed control schemes with sensory feedback of load information to reproduce the multi-legged locomotion observed when a part of the terrain is removed. They also investigated the transition between swimming and walking of centipedes based on the interplay of brain, CPG, and sensory feedback (Yasui et al., 2019). However, the mechanism for generating various interlimb coordination in multi-legged locomotion is still not fully understood.

The purpose of this study was to use a simple model to demonstrate that local sensory feedback generates the various interlimb coordination observed in multi-legged animals via embodied sensory-motor interactions. For that purpose, we constructed a mechanical model that imitates the flexible body of multi-legged animals and a control model that uses phase oscillators inspired by CPGs and phase resetting as local sensory feedback. The simulation results show that although the oscillators do not interact directly, three types of gaits (direct-, retrograde-, and source-wave gaits) emerge through the embodied sensorimotor interaction. Furthermore, we derive these analytical solutions and stabilities under some assumptions, which produce the three types of gaits regardless of the number of legs and clarify how the local sensory feedback generates these gaits through the embodied sensorimotor interaction.

## 2. Simulation

### 2.1. Mechanical Model

Skeletal structure and muscle arrangement have large effects on animal locomotion (Ting and Chiel, 2017), and appropriate models need to be created depending on the motion of interest. In particular, in fast locomotion where inertial effects are larger than viscous ones, body elasticity mainly determines the motion. For example, a spring-loaded inverted pendulum (SLIP) model has been widely used to investigate the characteristics of running in mammals (Full and Koditschek, 1999; Tanase et al., 2015; Adachi et al., 2020; Kamimura et al., 2021) and in cockroaches (Seipel et al., 2004; Spence et al., 2010). Detailed musculoskeletal models have been also used (Proctor and Holmes, 2018). Conversely, in slow locomotion, viscosity plays a dominant role. In stick insects, soon after swing muscle activity stops, swing leg movement ceases (Hooper et al., 2009). The effects of sensory feedback are dominant for slow-walking insects (Daun-Gruhn and Büschges, 2011).

**Figure 1:**
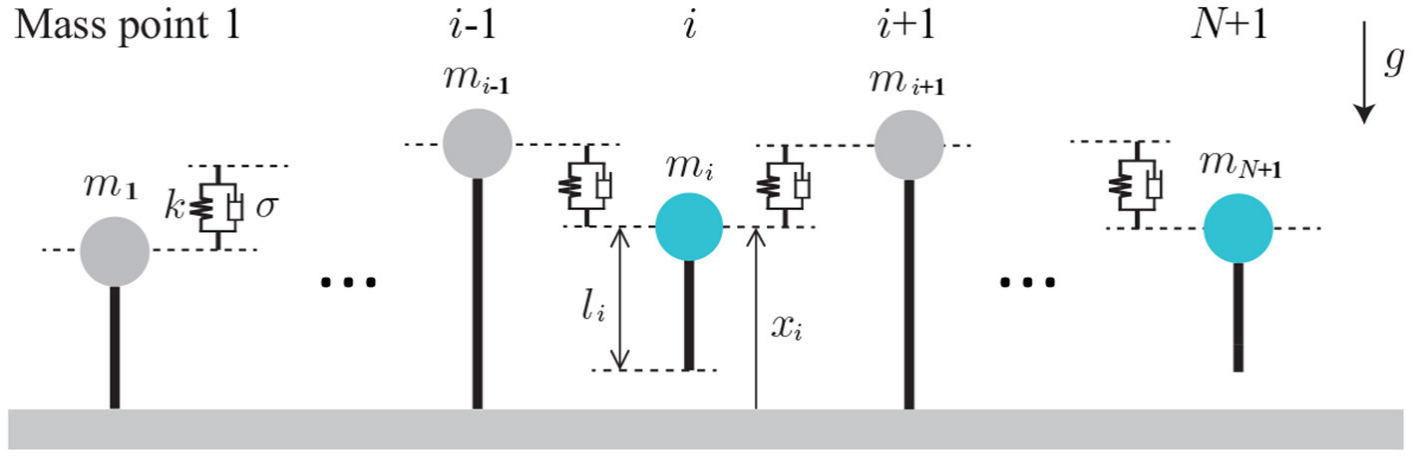
Mechanical model composed of (*N*+1) mass points. The mass points move only vertically and are connected via springs and dampers. Dotted lines represent the vertical positions of the mass points.

In this study, we focus on relatively slow locomotion to investigate the functional roles of sensory feedback in multi-legged locomotion. We construct a simple mechanical model with flexible body and legs. Specifically, the mechanical model is composed of (*N* + 1) mass points, whose mass is *m*_*i*_ and whose height is *x*_*i*_ (*i* = 1, …, *N* + 1), as shown in Figure 1. The mass points move only vertically and are connected by springs and dampers (all the spring constants are κ and the damper constants are σ). The neutral length of the spring is 0. Each mass point has a massless leg (Leg *i*), which also moves vertically and whose length is *l*_*i*_. The ground is modeled as a spring and damper (with spring constant *K* and damper constant *D*). The ground is much stiffer than the body spring (*K* ≫ κ) and the damper coefficient is set to provide overdamping. We used *m*_*i*_ = *m* for *i* = 2, …, *N* and *m*_1_ = *m*_*N*+1_ = *m*_B_ for the edges, where *m*/2 < *m*_B_ < *m*. The gravitational acceleration is *g*.

For *N* ≥ 3, the equations of motion are given by

(1) $$\begin{array}{l} m_{i}{\ddot{x}}_{i}=\\ \{\begin{array}{cc} \begin{array}{l} -\kappa (x_{1}-x_{2})-\sigma ({\dot{x}}_{1}-{\dot{x}}_{2})-m_{1}g\\ \;-p_{1}\left\{K(x_{1}-l_{1})+D({\dot{x}}_{1}-{\dot{l}}_{1})\right\} \end{array} & i=1\\ \begin{array}{l} -\kappa (2x_{i}-x_{i+1}-x_{i-1})-\sigma (2{\dot{x}}_{i}-{\dot{x}}_{i+1}-{\dot{x}}_{i-1})-m_{i}g\\ \;-p_{i}\left\{K(x_{i}-l)+D({\dot{x}}_{i}-{\dot{l}}_{i})\right\} \end{array} & i=2,\ldots ,N\\ \begin{array}{l} -\kappa (x_{N+1}-x_{N})-\sigma ({\dot{x}}_{N+1}-{\dot{x}}_{N})-m_{N+1}g\\ \;-p_{N+1}\left\{K(x_{N+1}-l_{N+1})+D({\dot{x}}_{N+1}-{\dot{l}}_{N+1})\right\} \end{array} & i=N+1 \end{array} \end{array}$$

where *p*_*i*_(*i* = 1, …, *N* + 1) represents whether Leg *i* is in contact with the ground and is defined by,

(2) $$\begin{array}{l} p_{i}=\{\begin{array}{cc} 0 & l_{i}<x_{i}\\ 1 & \text{otherwise}. \end{array} \end{array}$$

### 2.2. Control Model

To understand the mechanism that generates rhythmic leg movement, central pattern generators (CPGs) have been well studied (Ijspeert, 2008; Daun-Gruhn and Büschges, 2011) and many modeling approaches are available for CPGs, such as relatively detailed biophysical models based on Hodgkin-Huxley-type neuron models (Rybak et al., 2006; Daun et al., 2009), connectionist models composed of simplified neuron models (e.g., leaky-integrator neurons) (Ijspeert, 2001; Pasemann et al., 2003) and abstract models using van der Pol and Matsuoka oscillators (van der Pol, 1926; Matsuoka, 1987). In contrast, some studies proposed reflex chains in place of CPGs to generate rhythmic leg movement, such as Walknet (Cruse et al., 1998), and others proposed heteroclinic oscillator models to represent intermediate behavior between the CPGs and reflex chains (Shaw et al., 2015).

**Figure 2:**
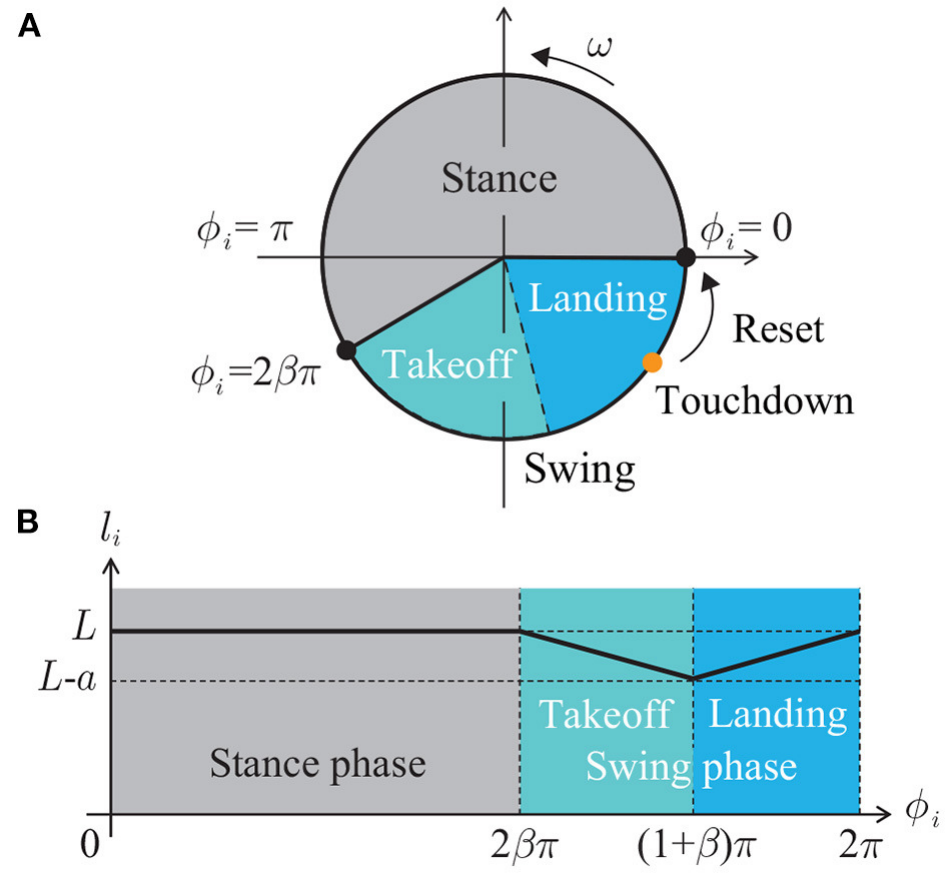
**(A)** Phase oscillator model. **(B)** Leg length *l*_*i*_ depending on phase ϕ_*i*_.

These models are nonlinear, interact with sensory feedback, and show remarkable adaptation to the environment and prominent replication of animal movement, such as entrainment to limit cycle and extending the timing of the state transition. However, these models are complicated to understand the functional role of embodied sensorimotor interaction for interlimb coordination. We use a simple phase oscillator model developed in our previous studies (Aoi et al., 2017; Ambe et al., 2018) for better understanding of the functional role through the analytical description. Specifically, each leg has a phase oscillator whose phase is ϕ_*i*_ ∈ [0, 2π) (mod 2π) (*i* = 1, …, *N*+1). The leg length *l*_*i*_ is determined by ϕ_*i*_ as follows (Figure 2):

(3) $$\begin{array}{l} l_{i}=l\left(\phi_{i}\right)=\{\begin{array}{cc} L & 0\leq \phi_{i}\leq 2\beta \pi \\ L-a\frac{\phi_{i}-2\beta \pi }{\left(1-\beta \right)\pi } & 2\beta \pi <\phi_{i}<\left(1+\beta \right)\pi \\ L-a\frac{2\pi -\phi_{i}}{\left(1-\beta \right)\pi } & \left(1+\beta \right)\pi \leq \phi_{i}<2\pi \end{array} \end{array}$$

where *L*, *a*(< *L*), and β ∈ (0.5, 1) are the maximum length, the amplitude of the swing leg movement, and the duty factor (ratio between the stance phase and step cycle durations), respectively. We defined the leg length by a piecewise-linear function and used *mg*/κ < *a* so that the leg is in the air in the swing phase (ϕ_*i*_ ∈ (2βπ, 2π)). While the length is *L* in the stance phase (ϕ_*i*_ ∈ [0, 2βπ]), it shortens in the takeoff phase (first half of the swing phase, ϕ_*i*_ ∈ (2βπ, (1+β)π)) and lengthens in the landing phase (second half of the swing phase, ϕ_*i*_ ∈ [(1 + β)π, 2π)). The oscillator phase follows the following dynamics:

(4) $$\begin{array}{l} \frac{d\phi_{i}}{dt}=\omega +y_{i}, \end{array}$$

where ω is the basic frequency of locomotion and *y*_*i*_ represents a sensory feedback. We used $\omega \ll \sqrt{\kappa /m_{i}}$ to make the model walk slowly.

Sensory feedback has been physiologically well studied in insects (Delcomyn, 2004; Büschges et al., 2008). Insects have mechanoreceptors that can sense various information, such as contact with the ground, joint angles, and mechanical load at the nearby leg joint (Tuthill and Wilson, 2016). Especially in stick insects, sensory feedback plays an important role for inter-joint coordination. Strain signals from the trochanter play a major role in shaping thorax-coxa (TC)-joint motoneuronal activity during walking and contribute to the coordination of the TC-joint movement with the stepping pattern of the distal leg joints (Akay et al., 2004). Centipedes also sense load information and stop their periodic leg movements when the terrain is removed (Yasui et al., 2017). Locomotion rhythm resetting and phase shifting in motorneuron activities by sensory feedback and perturbation (phase resetting) has been observed in insects (Büschges, 1995), as well as in mammals (Schomburg et al., 1998; Rybak et al., 2006). Because phase resetting is amenable to mathematical analysis to determine the essential functional role of sensory feedback, this study focuses on phase resetting based on load information. Based on our previous studies (Aoi et al., 2013; Ambe et al., 2015, 2018), we incorporated the phase resetting mechanism as

(5) $$\begin{array}{l} \tau \frac{dy_{i}}{dt}=-y_{i}+u_{i} \end{array}$$

(6) $$\begin{array}{l} u_{i}=\{\begin{array}{cc} 0 & 0\leq \phi_{i}<\left(1+\beta \right)\pi \\ \left(2\pi -\phi_{i}\right)\delta \left(t-t_{i}^{i}\right) & \left(1+\beta \right)\pi \leq \phi_{i}<2\pi \end{array} \end{array}$$

where $t_{i}^{i}$ is the time when Leg *i* touches the ground and δ() is Dirac's delta function. When Leg *i* touches the ground in the landing phase ((1 + β)π ≤ ϕ_*i*_ < 2π), the phase ϕ_*i*_ is reset to zero as shown in Figure 2A. In the present study, we used a first-order lag system with time constant τ to change the phase value continuously after the phase resetting for the simulation.

Because the leg movements of our model are determined by the oscillation phases, the relative phases between the oscillators ψ_*i*_ ∈ [0, 2π) (mod 2π)(*i* = 1, …, *N*) explain the gait, which is given by

(7) $$\begin{array}{l} \psi_{i}=\phi_{i+1}-\phi_{i}. \end{array}$$

We investigated where the relative phases converged through the mechanical dynamics (1) and phase dynamics (4).

### 2.3. Results

We conducted forward dynamic simulations of our model in case of *N* = 3 to find stable gaits. All simulations used the parameters in Table 1. To investigate the characteristics of the gaits, we defined a Poincaré section Σ^1^ for the relative phases just before Leg 1 touches the ground, where we used $\Psi^{1}\equiv {\left[\psi_{1}^{1}\psi_{2}^{1}\psi_{3}^{1}\right]}^{\text{T}}$ and ()^*i*^ represents the value just before Leg *i* touches the ground.

**Table 1:** Parameters for simulation.

**Parameter**	**Value**	**Parameter**	**Value**	**Parameter**	**Value**
*m*_B_ [kg]	0.90	κ [N/m]	100	ω [rad/s]	0.4 $\ll \sqrt{\kappa /m}$
*m* [kg]	1.0	σ [Ns/m]	$2\sqrt{\kappa }$	*g* [m/s^2^]	9.8
*L* [m]	1.0	*K* [N/m]	10^5^	τ	0.5
*a* [m]	0.4	*D* [Ns/m]	$2\sqrt{K}$	β	0.8

#### 2.3.1. Periodic Solutions

**Figure 3:**
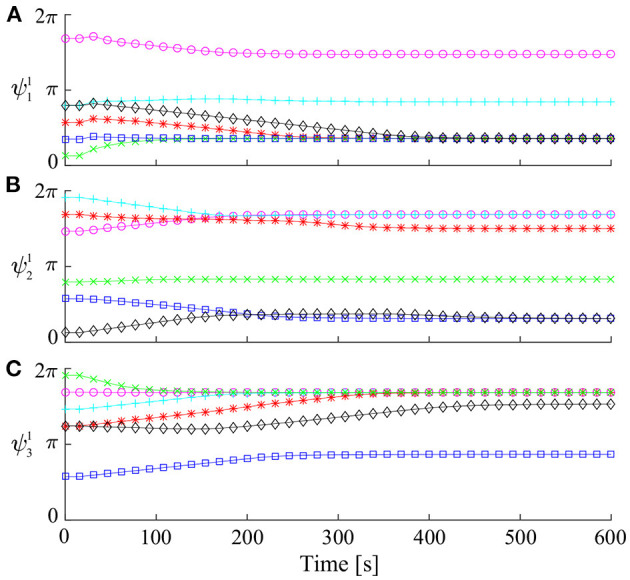
Time profile of relative phases for **Ψ**^1^ simulated from six sets of initial values: **(A)** $\psi_{1}^{1}$, **(B)** $\psi_{2}^{1}$, and **(C)** $\psi_{3}^{1}$.

First, we used six sets of the initial relative phases for **Ψ**^1^ to investigate if and how they converged. Figure 3 shows the time profile of the relative phases on Σ^1^ for the six sets of the initial relative phases. Some of the relative phases converged to identical values, but others converged to different values. These converged relative phases correspond to periodic solutions. These results suggest that there are many periodic solutions.

**Figure 4:**
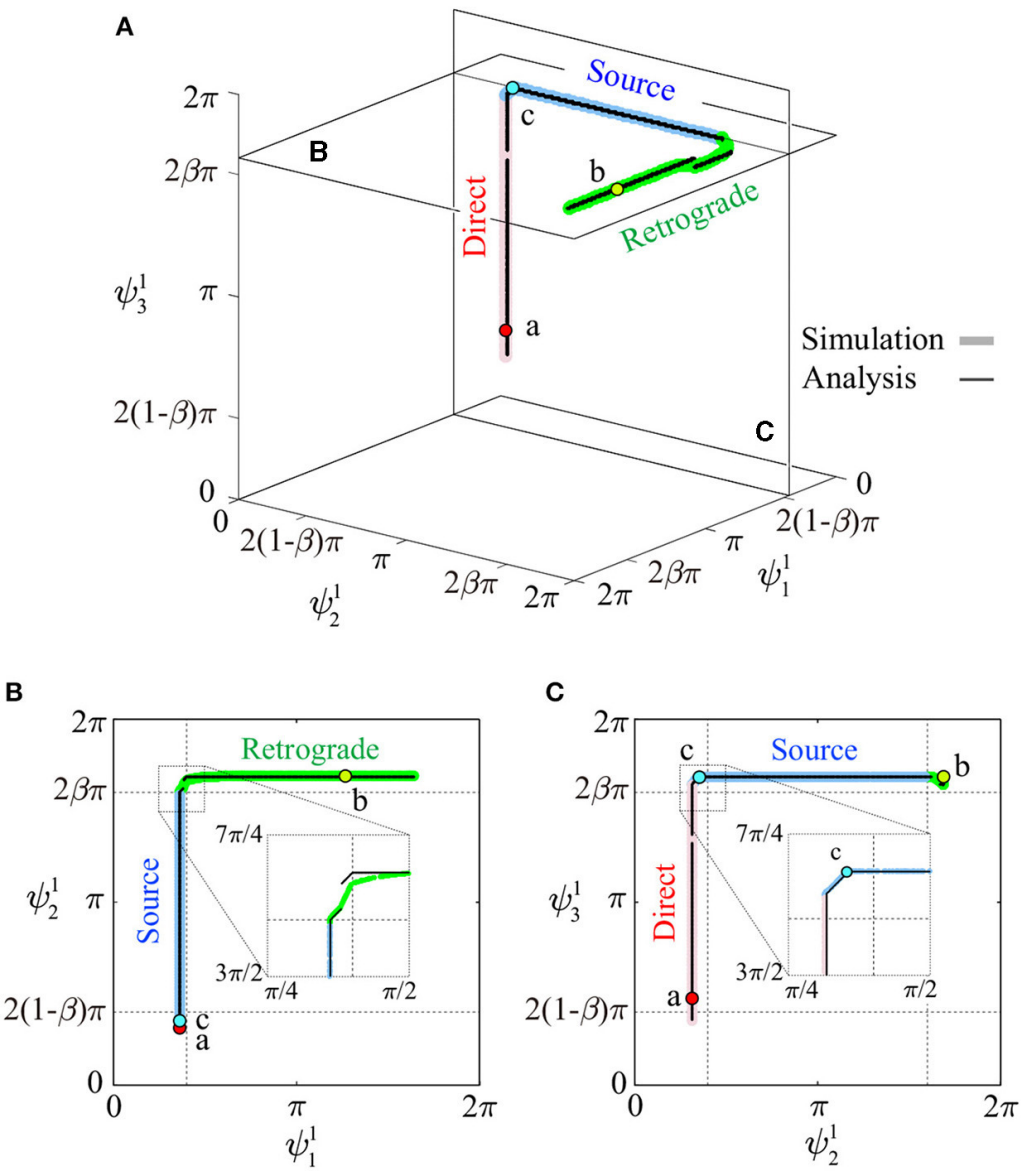
Relative phases for **Ψ**^1^ of periodic solutions: **(A)** shown in $\psi_{1}^{1}$-$\psi_{2}^{1}$-$\psi_{3}^{1}$ space, **(B)** projected to a plane parallel to the $\psi_{1}^{1}$-$\psi_{2}^{1}$ plane, and **(C)** projected to a plane parallel to the $\psi_{2}^{1}$-$\psi_{3}^{1}$ plane. Thick colored lines and thin black lines represent simulated and analytic solutions, respectively. Points a, b, and c are used to show footprint diagrams in Figure 6.

To illustrate details of the periodic solutions, Figure 4A uses thick colored lines (red, blue, and green) to show the relative phases converged from many initial values (25 × 25 × 25) for **Ψ**^1^. The solutions generally consist of three connected orthogonal line segments and lay close to two planes parallel to the $\psi_{1}^{1}$–$\psi_{2}^{1}$ and $\psi_{2}^{1}$–$\psi_{3}^{1}$ planes. Figures 4B,C show the solutions projected onto each plane. Near the connections between line segments, the segments are slightly bent. The three line segments correspond to the source-wave, retrograde-wave, and direct-wave gaits, as investigated below. The thin black line segments represent the analytical approximate solutions obtained in section 3.

**Figure 5:**
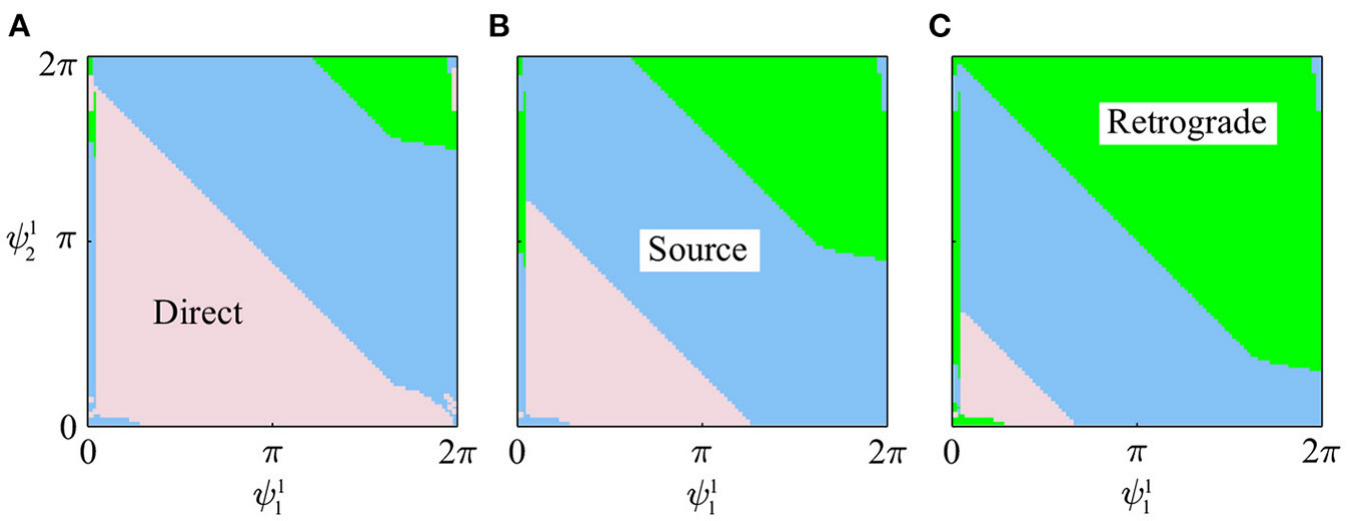
Slices of basin of attraction for three gaits in simulation under three conditions: **(A)** $\psi_{3}^{1}=2\left(1-\beta \right)\pi$, **(B)** $\psi_{3}^{1}=\pi$, and **(C)** $\psi_{3}^{1}=2\beta \pi$.

Figures 5A–C show the slices of basin of attraction under the conditions $\psi_{3}^{1}=2\left(1-\beta \right)\pi ,\pi$, and 2βπ, respectively. All the initial values (99 × 99) converged to one of the three gaits, and the basin of attraction was separated into three parts, each corresponding to one gait type.

#### 2.3.2. Characteristics of Three Types of Gaits

**Figure 6:**
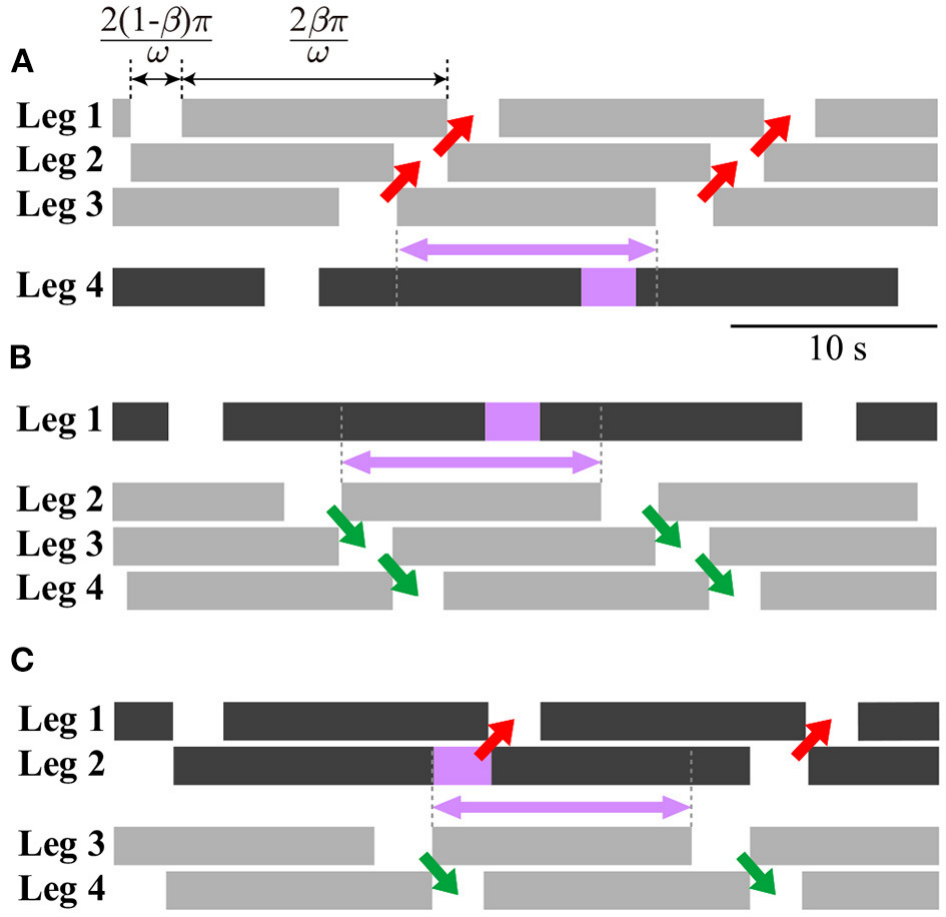
Footprint diagrams of periodic solutions: **(A)** Direct-wave gait (point a in Figure 4), **(B)** retrograde-wave gait (point b in Figure 4), and **(C)** source-wave gait (point c in Figure 4). Black bars can change to gray bars as long as the violet box is in the range indicated by the violet arrow.

The obtained periodic solutions were categorized by three gaits: direct-, retrograde-, and source-wave gaits. The direct-wave gait has the following relative phases:

$$\begin{array}{l} {\hat{\psi }}_{1}^{1}\approx 2\left(1-\beta \right)\pi ,{\hat{\psi }}_{2}^{1}\approx 2\left(1-\beta \right)\pi ,2\left(1-\beta \right)\pi ⪅{\hat{\psi }}_{3}^{1}⪅2\beta \pi \end{array}$$

where ()^ indicates the periodic solution. This gait appears as one line segment, as shown in Figure 4. Figure 6A shows the footprint diagram for ${\hat{\Psi }}^{1}={\left[1.13\;0.98\;1.50\right]}^{\text{T}}$ (point a in Figure 4). In this gait, the swing leg movement of Legs 1, 2, and 3 propagates from posterior to anterior with the same interval, as shown by red arrows. However, the relative phase of the swing leg movement between Legs 3 and 4 can vary within the range indicated by the violet arrow.

The retrograde-wave gait has the following relative phases:

$$\begin{array}{l} 2\left(1-\beta \right)\pi ⪅{\hat{\psi }}_{1}^{1}⪅2\beta \pi ,{\hat{\psi }}_{2}^{1}\approx 2\beta \pi ,{\hat{\psi }}_{3}^{1}\approx 2\beta \pi . \end{array}$$

This also appears as one line segment (to be precise, it is slightly curved) in Figure 4. Figure 6B shows the footprint diagram for ${\hat{\Psi }}^{1}={\left[3.97\;5.29\;5.29\right]}^{\text{T}}$ (point b in Figure 4). In this gait, the swing leg movement of Legs 2, 3, and 4 propagates from anterior to posterior with the same interval, as shown by green arrows. However, the relative phase of the swing leg movement between Legs 1 and 2 can vary in the range indicated by the violet arrow.

The source-wave gait has the following relative phases:

$$\begin{array}{l} {\hat{\psi }}_{1}^{1}\approx 2\left(1-\beta \right)\pi ,2\left(1-\beta \right)\pi ⪅{\hat{\psi }}_{2}^{1}⪅2\beta \pi ,{\hat{\psi }}_{3}^{1}\approx 2\beta \pi . \end{array}$$

This also appears as one line segment (to be precise, it comprises two line segments) in Figure 4. Figure 6C shows the footprint diagram for **Ψ**^1^ = [1.13 1.10 5.28]^T^ (point c in Figure 4). In this gait, the swing leg movement of Legs 1 and 2 propagates from posterior to anterior, as shown by red arrows, while that of Legs 3 and 4 propagates from anterior to posterior as shown by green arrows. However, the relative phase of the swing leg movement between Legs 2 and 3 can vary in the range indicated by the violet arrow.

These three types of gaits were not predetermined, but emerged through the mechanical and sensory interaction. These gaits are represented by line segments for **Ψ**^1^, which are serially connected as shown in Figure 4.

## 3. Analysis

**Figure 7:**
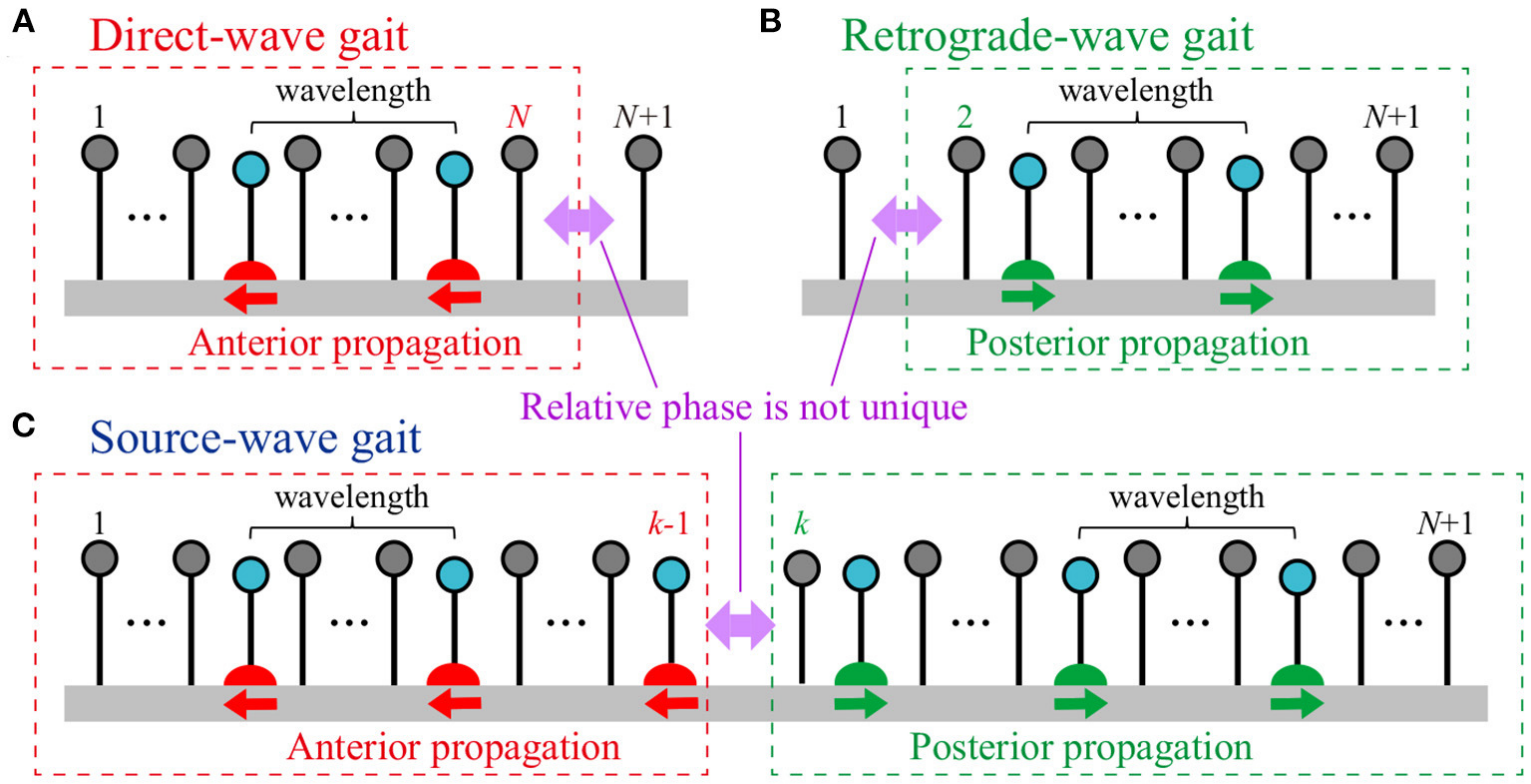
Three types of gaits for *N*+1 mass points: **(A)** Direct-wave, **(B)** retrograde-wave, and **(C)** source-wave gaits. In the direct-wave (retrograde-wave) gait, while the swing leg movements of Legs 1 to *N* (Legs 2 to *N*+1) propagate anteriorly (posteriorly) with a constant relative phase, the relative phase between Legs *N* and *N* + 1 (Legs 1 and 2) is not unique. In the source-wave gait, while the swing leg movements of the anterior part (Legs 1 to *k*−1, *k* ∈ [2, *N*]) propagate anteriorly, those of the posterior part (Legs *k* to *N* + 1) propagate posteriorly. The relative phase between Legs *k*−1 and *k* is not unique. In these three gaits, there is only one swing leg within one wavelength.

Three types of gaits were obtained via the mechanical and sensory interaction in simulations with a multi-mass spring model. Although the models described in section 2 used four mass points, these gaits are expected to appear in models with more mass points (attached Supplementary Video 1). Figure 7 shows the three types of gaits for *N*+1 mass points. For the direct-wave gait (Figure 7A), the swing leg movements propagate anteriorly, where there is only one swing leg within one wavelength. While the phase difference between the neighboring legs for Legs 1 to *N* is around 2(1−β)π, that between Legs *N* and *N*+1 is not unique and is variable from 2(1−β)π to 2βπ. For the retrograde-wave gait (Figure 7B), the swing leg movements propagate posteriorly, where there is only one swing leg within one wavelength. While the phase difference between the neighboring legs for Legs 2 to *N* + 1 is around 2βπ, that between Legs 1 and 2 is not unique and is variable from 2(1 − β)π to 2βπ. For the source-wave gait (Figure 7C), while the anterior swing leg movements (Legs 1 to *k* − 1, *k* ∈ [2, *N*]) propagate anteriorly, those of the posterior part (Legs *k* to *N* + 1) propagate posteriorly. Although the phase differences of the neighboring legs for the anterior and posterior parts are around 2(1 − β)π and 2βπ, respectively, that between Legs *k* − 1 and *k* is not unique and is variable from 2(1 − β)π to 2βπ. To clarify the mechanism for generating these three gaits, we simplified our model by some physical assumptions to derive analytical solutions.

A concrete way to derive the analytical solutions is as follows. First, we simplify our mechanical and controller models. In particular, we assume that the natural frequencies of the mass points are larger than the gait frequency so that the vertical position of the mass points *x*_*i*_ (*i* = 1, …, *N* + 1) is determined uniquely by the oscillator phase ϕ_*i*_ in section 3.1. We also assume that the time constant of our control model is smaller than the gait cycle duration found in section 3.2, which allows the Poincaré map to be obtained by determining the phase resetting value $\phi_{i}^{i}$. These assumptions give a touchdown relation between $\phi_{i}^{i},\phi_{i-1}^{i}$, and $\phi_{i+1}^{i}$. In section 3.3, we determine the order of the touchdown events in the case of the source-wave gait based on the simulation results and rewrite the touchdown relation by $\phi_{i-1}^{i-1},\phi_{i}^{i},\phi_{i+1}^{i+1}$, and the relative phases between the oscillators, which gives periodic solutions of the source-wave gait. In section 3.4, we investigate the stability of the periodic solutions. In section 3.5, we derive the periodic solutions and stability of the direct-wave gait from the front–rear symmetry. In section 3.6, we compare the analytical results with the simulation results. In section 3.7, we describe the geometrical meaning of the obtained solutions.

### 3.1. Simplification of Mechanical Model

We used $\omega \ll \sqrt{\kappa /m_{i}}$ in (4) to make the model walk slowly in the simulation. Thus, we ignored the dynamics of the mass points and focused only on the equilibrium of forces by replacing the ground reaction forces with unknown variables. Then, (1) becomes

(8) $$\begin{array}{l} \{\begin{array}{cc} \kappa \left(x_{2}-x_{1}\right)+R_{1}=m_{1}g & i=1\\ \kappa \left(x_{i-1}-x_{i}\right)+\kappa \left(x_{i+1}-x_{i}\right)+R_{i}=m_{i}g & i\in \left[2,N\right]\\ \kappa \left(x_{N}-x_{N+1}\right)+R_{N+1}=m_{N+1}g & i=N+1 \end{array}, \end{array}$$

where *R*_*i*_ is the ground reaction force. The number of unknown variables is 2(*N* + 1); *x*_1_, …, *x*_*N*+1_ and *R*_1_, …, *R*_*N*+1_. These variables depend on the foot contact conditions. In particular, when Leg *i* is in the air, *x*_*i*_ ≥ *l*(ϕ_*i*_) and *R*_*i*_ = 0. In contrast, when Leg *i* is in contact with the ground, *x*_*i*_ = *l*(ϕ_*i*_) and *R*_*i*_ > 0. Therefore, when ϕ_*i*_ and foot contact conditions for all legs are given, the number of the unknown variables is reduced to *N* + 1 and the unknown variables are solved by (8).

### 3.2. Simplification of Control Model

Although we used a first-order lag system in (9) for the simulation, the time constant τ is small compared with the gait period. Therefore, we set τ = 0, which reduces (4) to

(9) $$\begin{array}{l} \frac{d\phi_{i}}{dt}=\omega +u_{i}. \end{array}$$

This gives

(10) $$\begin{array}{l} \frac{d\psi_{i}}{dt}=u_{i+1}-u_{i}i=1,\ldots ,N. \end{array}$$

A Poincaré section Σ^*j*^ (*j* = 1, …, *N*+1) was defined for the relative phases just before the touchdown of Leg *j* and we used $\Psi^{j}\equiv {\left[\psi_{1}^{j}\psi_{2}^{j}\ldots \psi_{N}^{j}\right]}^{\text{T}}$. The Poincaré map for Σ^*j*^ was given by integrating (10) for one period. Under the assumption that each leg experiences phase resetting once each period, the map is given by

(11) $$\begin{array}{l} \psi_{i}^{j}\mapsto \psi_{i}^{j}+\phi_{i}^{i}-\phi_{i+1}^{i+1}i=1,\ldots ,N. \end{array}$$

To obtain the Poincaré map, $\phi_{i}^{i}\left(i=1,\ldots ,N+1\right)$ needs to be determined.

Based on the periodic solutions, we assume that there is only one swing leg within one wavelength, just before Leg *i* touches the ground (*i* = 1, …, *N*+1), the neighboring legs (Legs *i*−1 and *i*+1) are in contact with the ground. Therefore, $x_{i-1}=l\left(\phi_{i-1}^{i}\right)$, $x_{i}=l\left(\phi_{i}^{i}\right)$, $x_{i+1}=l\left(\phi_{i+1}^{i}\right)$, *R*_*i*−1_ > 0, *R*_*i*_ = 0, and *R*_*i*+1_ > 0 are satisfied just before the touchdown of Leg *i*. By substituting these variables into (8), we obtain

(12) $$\begin{array}{l} l\left(\phi_{i}^{i}\right)=\{\begin{array}{cc} f_{\text{B}}\left(\phi_{2}^{i}\right) & i=1\\ f\left(\phi_{i-1}^{i},\phi_{i+1}^{i}\right) & i\in \left[2,N\right]\\ f_{\text{B}}\left(\phi_{N}^{i}\right) & i=N+1 \end{array}, \end{array}$$

where

(13) $$\begin{array}{l} f\left(\phi_{\text{h}},\phi_{\text{f}}\right)=\frac{1}{2}\left\{l\left(\phi_{\text{h}}\right)+l\left(\phi_{\text{f}}\right)\right\}-\frac{mg}{2\kappa } \end{array}$$

$$\begin{array}{l} f_{\text{B}}\left(\phi \right)=l\left(\phi \right)-\frac{m_{\text{B}}g}{\kappa }. \end{array}$$

The substitution of (12) into (3) gives one relation between $\phi_{i-1}^{i},\phi_{i}^{i}$, and $\phi_{i+1}^{i}$ for each *i* (*i* = 1, …, *N* + 1). Because we can write $\phi_{i-1}^{i}$ and $\phi_{i+1}^{i}$ as $\phi_{i-1}^{i-1},\phi_{i}^{i},\phi_{i+1}^{i+1}$, and **Ψ**^*j*^, as discussed in the following section, $\phi_{i}^{i}$ is obtained by **Ψ**^*j*^. As a result, we obtain the Poincaré map from (11).

### 3.3. Analytical Solution of Source-Wave Gait

Here, we derive the periodic solution of the source-wave gait characterized by Leg *k* ∈ [2, *N*] that determines the boundary between the direct- and retrograde-wave regions (Figure 7C). The solution of the source-wave gait is useful for deriving those of the direct- and retrograde-wave gaits. In particular, the solution of the retrograde-wave gait is obtained by *k* = 2 for the solution of the source-wave gait (Figure 7B). However, note that *k* = *N*+1 does not fully explain the solution of the direct-wave gait, which is instead derived by the front–rear symmetry and the solution of the retrograde-wave gait, as explained in section 3.5.

To derive the periodic solution of the source-wave gait, we first define the Poincaré section as Σ^*k*^ and transform the right-hand side of (12) to be represented by the relative phases **Ψ**^*k*^ and the touchdown phase $\phi_{i}^{i}$ (*i* = 1, …, *N* + 1). For that purpose, we assume some conditions for the phases of the neighboring legs at each touchdown event, determine the order of the touchdown events, and represent the relative phases of the neighboring legs of the touchdown leg using **Ψ**^*k*^ and $\phi_{i}^{i}$ by accounting for the effects of phase resetting. Second, we derive the conditions of $\phi_{i}^{i}$ for the periodicity. The reduced equation of (12) and the periodicity conditions yield the periodic solution.

**Figure 8:**
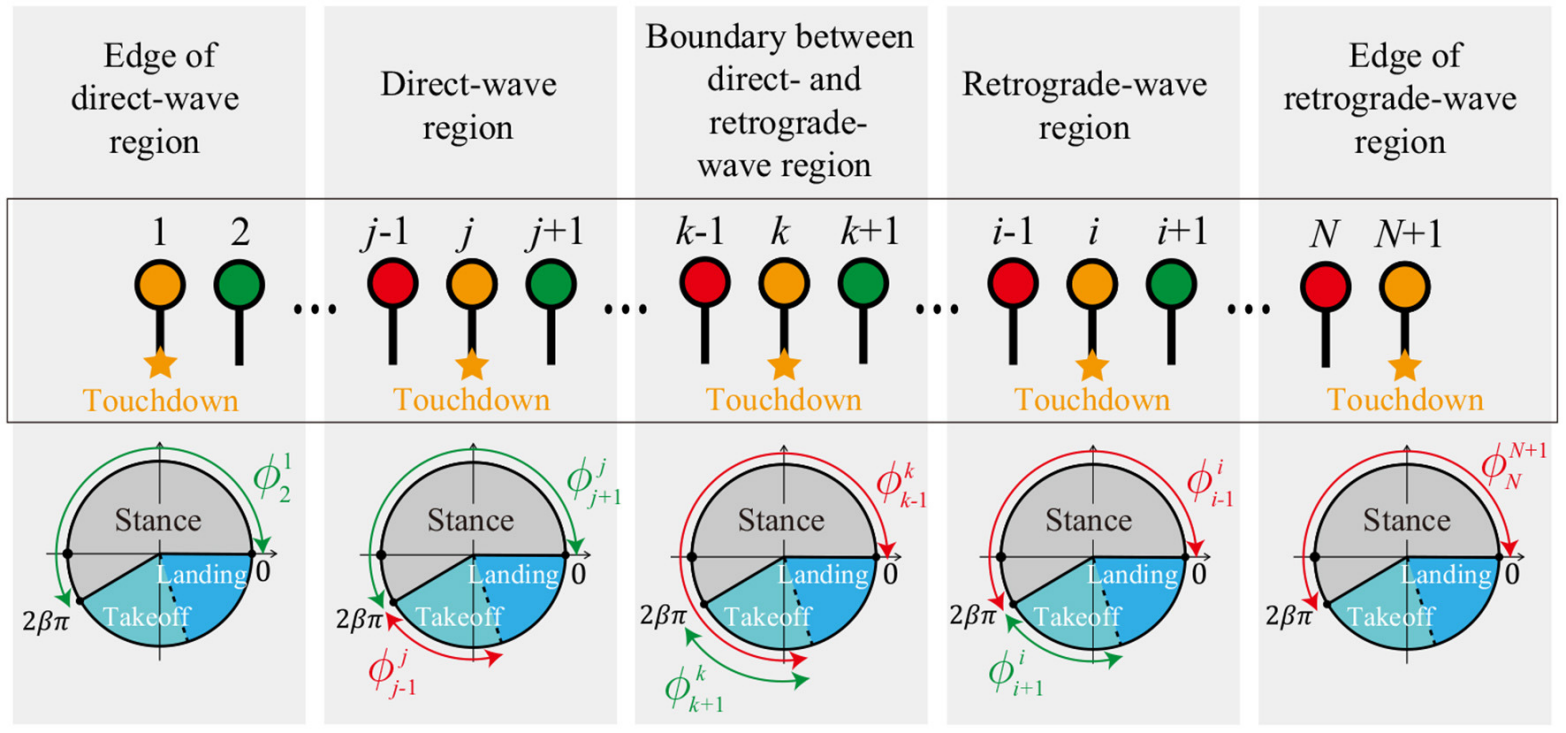
Assumptions for the phases of the neighboring legs at each touchdown event for the source-wave gait.

Based on the simulation results obtained in section 2, we assume the following conditions for the phases of the neighboring legs at each touchdown event (Figure 8). For the touchdown of Leg *k* (boundary between direct- and retrograde-wave regions), $\phi_{k-1}^{k}$ is in the stance or takeoff phase ($0\leq \phi_{k-1}^{k}<\left(1+\beta \right)\pi$), and $\phi_{k+1}^{k}$ is in the takeoff phase ($2\beta \pi \leq \phi_{k+1}^{k}<\left(1+\beta \right)\pi$). For the touchdown of Leg *j* ∈ [2, *k* − 1] (direct-wave region), $\phi_{j-1}^{j}$ is in the takeoff phase ($2\beta \pi \leq \phi_{j-1}^{j}<\left(1+\beta \right)\pi$) and $\phi_{j+1}^{j}$ is in the stance phase ($0\leq \phi_{j+1}^{j}<2\beta \pi$) because the swing movements of Legs 1 to *k* − 1 propagate anteriorly. For the touchdown of Leg *i* ∈ [*k* + 1, *N*] (retrograde-wave region), $\phi_{i-1}^{i}$ is in the stance phase ($0\leq \phi_{i-1}^{i}<2\beta \pi$) and $\phi_{i+1}^{i}$ is in the takeoff phase ($2\beta \pi \leq \phi_{i+1}^{i}<\left(1+\beta \right)\pi$) because the swing movements of Legs *k* to *N* + 1 propagate posteriorly. For the touchdown of Legs 1 and *N* + 1 (edges of direct- and retrograde-wave regions), $\phi_{2}^{1}$ and $\phi_{N}^{N+1}$ are in the stance phases ($0\leq \phi_{2}^{1},\phi_{N}^{N+1}<2\beta \pi$).

**Figure 9:**
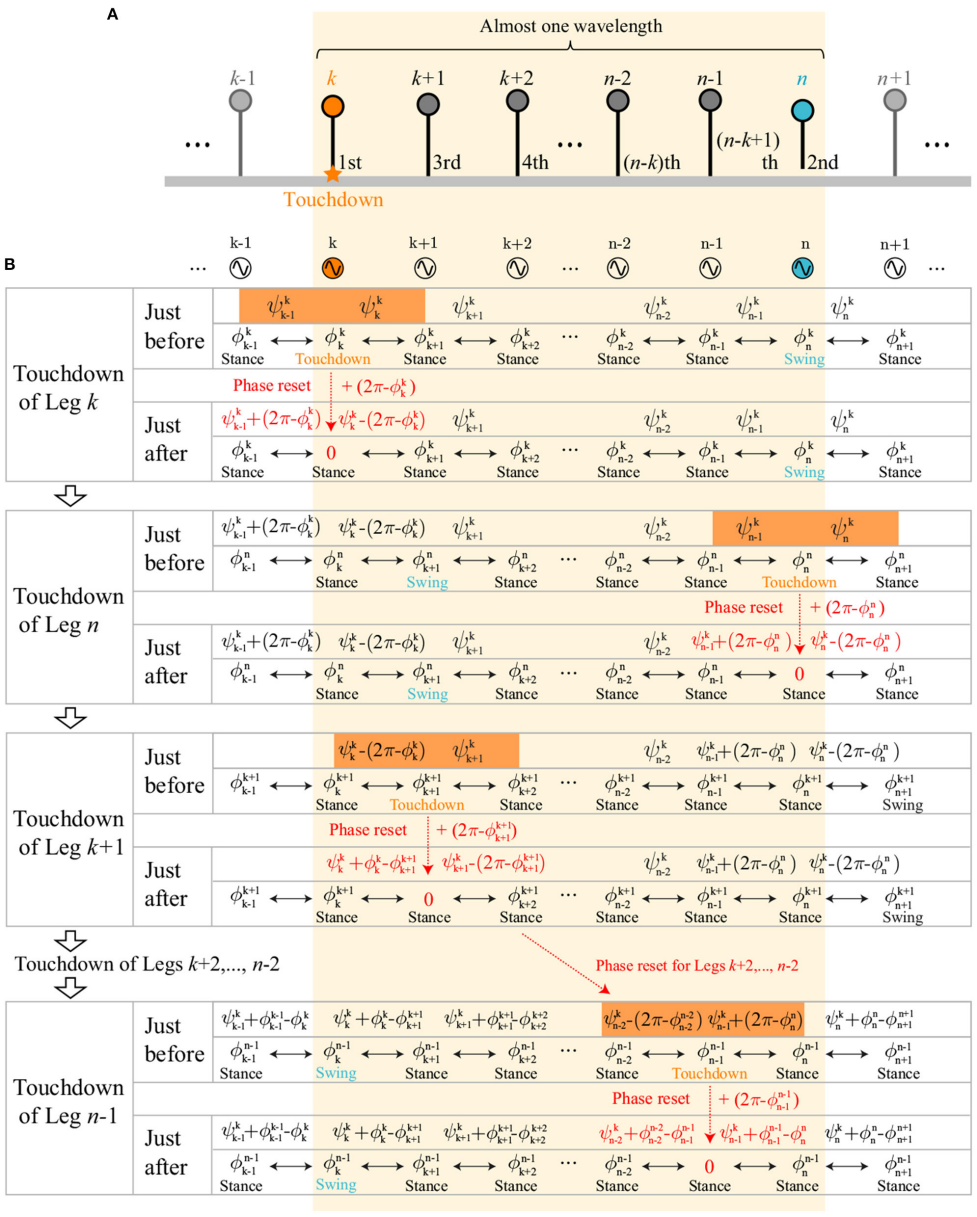
Evolution of oscillator phases within one wavelength: **(A)** Order of touchdown events of Legs *k* to *n* in the retrograde-wave region. Legs *k*, *n*, *k*+1, *k*+2, *k*+3, … , *n*−1 touch down sequentially. **(B)** Evolution of oscillator phases and relative phases at touchdown events of Legs *k* to *n*. Phase resetting at each touchdown changes only the oscillator phase of the touchdown leg and the relative phases of the neighboring legs. $\psi_{i-1}^{i}$ and $\psi_{i}^{i}$ just before the touchdown of Leg *i* (*i* = *k, n, k*+1, *k*+2, …, *n*−2, *n*−1) are represented by **Ψ**^*k*^, $\phi_{i-1}^{i-1}$, and $\phi_{i+1}^{i+1}$, as highlighted in orange.

These assumptions determine the order of touchdown events. In the retrograde-wave region, suppose that *n*(> *k*) is the minimum value that satisfies $\phi_{n}^{k}\in \left(\phi_{n}^{n-1},\phi_{n}^{n}\right]$, which means that Legs (*n*−1), *k*, and *n* touch the ground sequentially (Figure 9A). Legs *k* to *n* correspond to almost one wavelength of the gait. Because the swing movement propagates posteriorly from Legs *k* to *N*−1, Leg *n* is in the swing phase when Leg *k* touches down. Thus, the order of touchdown events for Legs *k* to *n* is determined as being in the order *k*, *n*, *k*−1, *k*−2, *k*−3, … , *n*−1, as shown in Figure 9A. The order of touchdown events in the direct-wave region is determined similarly.

When the order of touchdown events is determined, the relative phases $\psi_{i-1}^{i}$ and $\psi_{i}^{i}$ just before touchdown of Leg *i* can be represented using **Ψ**^*k*^ and $\phi_{i-1}^{i-1}$, and $\phi_{i+1}^{i+1}$ by accounting for the effects of phase resetting. Specifically, because phase resetting at each touchdown changes only the oscillator phase of the touchdown leg and the relative phases of the neighboring legs, $\psi_{i-1}^{i}$ and $\psi_{i}^{i}$ just before touchdown of Leg *i* can be represented using **Ψ**^*k*^, $\phi_{i-1}^{i-1}$, and $\phi_{i+1}^{i+1}$, depending on the experiences of the leg touchdowns. In the case of the retrograde-wave region, the evolution of the relative phases for Legs *k* to *n* at each touchdown event can be written as Figure 9B. For *i* = *k* and *n*, $\psi_{i-1}^{i}$ and $\psi_{i}^{i}$ are given by $\psi_{i-1}^{i}=\psi_{i-1}^{k}$ and $\psi_{i}^{i}=\psi_{i}^{k}$, respectively (highlighted in orange at the touchdowns of Legs *k* and *n* in Figure 9B), because both neighboring legs do not experience phase resetting in the period between the touchdown events of Legs *k* and *n*. As a result, $\phi_{i-1}^{i}=\phi_{i}^{i}-\psi_{i-1}^{k}$ and $\phi_{i+1}^{i}=\phi_{i}^{i}+\psi_{i}^{k}-2\pi$ ($\phi_{i+1}^{i}$ is subtracted by 2π to satisfy $0\leq \phi_{i+1}^{i}<2\pi$) are satisfied because $\psi_{i-1}^{i}=\phi_{i}^{i}-\phi_{i-1}^{i}$ and $\psi_{i}^{i}=\phi_{i+1}^{i}-\phi_{i}^{i}$. For *i* ∈ [*k*+1, *n*−2], $\psi_{i-1}^{i}$ and $\psi_{i}^{i}$ are given by $\psi_{i-1}^{i}=\psi_{i-1}^{k}-\left(2\pi -\phi_{i-1}^{i-1}\right)$ and $\psi_{i}^{i}=\psi_{i}^{k}$, respectively (highlighted in orange at the touchdown of Leg *k*+1 for *i* = *k*+1 in Figure 9B), because only the posterior Leg *i*−1 experiences phase resetting in the period between the touchdown events of Legs *k* and *i*. As a result, $\phi_{i-1}^{i}=\phi_{i}^{i}-\psi_{i-1}^{k}+2\pi -\phi_{i-1}^{i-1}$ and $\phi_{i+1}^{i}=\phi_{i}^{i}+\psi_{i}^{k}-2\pi$ are satisfied. For *i* = *n*−1, $\psi_{i-1}^{i}$ and $\psi_{i}^{i}$ are given by $\psi_{i-1}^{i}=\psi_{i-1}^{k}-\left(2\pi -\phi_{i-1}^{i-1}\right)$ and $\psi_{i}^{i}=\psi_{i}^{k}+\left(2\pi -\phi_{i+1}^{i+1}\right)$, respectively (highlighted in orange at the touchdown of Leg *n*−1 in Figure 9B), because both neighboring legs experience phase resetting in the interval between the touchdown events of Legs *k* and *n*−1. As a result, $\phi_{i-1}^{i}=\phi_{i}^{i}-\psi_{i-1}^{k}+2\pi -\phi_{i-1}^{i-1}$ and $\phi_{i+1}^{i}=\phi_{i}^{i}+\psi_{i}^{k}-\phi_{i+1}^{i+1}$ are satisfied. This means that $\phi_{i-1}^{i}$ and $\phi_{i+1}^{i}$ can be represented by **Ψ**^*k*^, $\phi_{i-1}^{i-1}$, $\phi_{i}^{i}$, and $\phi_{i+1}^{i+1}$. These analyses are also applicable to the direct-wave region.

By using these results, the right-hand side of (12) can be rewritten using $\psi_{i}^{k}\left(i=1,\ldots ,N\right)$ and $\phi_{i}^{i}\left(i=1,\ldots ,N+1\right)$ as

(14) $$\begin{array}{l} l\left(\phi_{i}^{i}\right)=\left\{ \begin{array}{cc} f_{\text{B}}\left(\phi_{1}^{1}+\psi_{1}^{k}-2\pi \right) & i\in S_{1}^{k}\\ f_{\text{B}}\left(\phi_{1}^{1}+\psi_{1}^{k}-\phi_{2}^{2}\right) & i\in S_{2}^{k}\\ f\left(\phi_{i}^{i}-\psi_{i-1}^{k},\phi_{i}^{i}+\psi_{i}^{k}-2\pi \right) & i\in S_{3}^{k}\\ f\left(\phi_{i}^{i}-\psi_{i-1}^{k}+2\pi -\phi_{i-1}^{i-1},\phi_{i}^{i}+\psi_{i}^{k}-\phi_{i+1}^{i+1}\right) & i\in S_{4}^{k}\\ f\left(\phi_{i}^{i}-\psi_{i-1}^{k},\phi_{i}^{i}+\psi_{i}^{k}-\phi_{i+1}^{i+1}\right) & i\in S_{5}^{k}\\ f\left(\phi_{i}^{i}-\psi_{i-1}^{k}+2\pi -\phi_{i-1}^{i-1},\phi_{i}^{i}+\psi_{i}^{k}-2\pi \right) & i\in S_{6}^{k}\\ f_{\text{B}}\left(\phi_{N+1}^{N+1}-\psi_{N}^{k}\right) & i\in S_{7}^{k}\\ f_{\text{B}}\left(\phi_{N+1}^{N+1}-\psi_{N}^{k}+2\pi -\phi_{N}^{N}\right) & i\in S_{8}^{k} \end{array}\right. \end{array}$$

where $S_{1-8}^{k}$ are classified based on whether the neighboring legs (Legs *i* − 1 and *i* + 1) of Leg *i* experience phase resetting in the interval between the touchdown events of Legs *k* and *i*, as shown in Table 2. Specifically, $S_{1,2,7,8}^{k}$ are for *i* = 1 and *N* + 1, which are given by

$$\begin{array}{l} S_{1}^{k}=\left\{i|i=1\right\}\cap \left\{i|\phi_{i}^{k}\in \left(\phi_{i}^{i+1},\phi_{i}^{i}\right]\right\}\\ S_{2}^{k}=\left\{i|i=1\right\}\cap \left\{i|i\notin S_{1}^{k}\right\}\\ S_{7}^{k}=\left\{i|i=N+1\right\}\cap \left\{i|\phi_{i}^{k}\in \left(\phi_{i}^{i-1},\phi_{i}^{i}\right]\right\}\\ S_{8}^{k}=\left\{i|i=N+1\right\}\cap \left\{i|i\notin S_{7}^{k}\right\}, \end{array}$$

$S_{3,4,5,6}^{k}$ are for *i* = 2 to *N*, which are given by

$$\begin{array}{l} S_{3}^{k}=\left\{i|i=k\right\}\cup \left\{i\in \left[2,k-1\right]|\phi_{i}^{k}\in \left(\phi_{i}^{i+1},\phi_{i}^{i}\right]\right\}\\ \;\cup \left\{i\in \left[k+1,N\right]|\phi_{i}^{k}\in \left(\phi_{i}^{i-1},\phi_{i}^{i}\right]\right\}\\ S_{4}^{k}=\left\{i\in \left[2,k-1\right]|\phi_{i-1}^{k}\in \left(\phi_{i-1}^{i},\phi_{i-1}^{i-1}\right]\right\}\\ \;\cup \left\{i\in \left[k+1,N\right]|\phi_{i+1}^{k}\in \left(\phi_{i+1}^{i},\phi_{i+1}^{i+1}\right]\right\}\\ S_{5}^{k}=\left\{i\in \left[2,k-1\right]|i\notin S_{3}^{k}\text{ and }i\notin S_{4}^{k}\right\}\\ S_{6}^{k}=\left\{i\in \left[k+1,N\right]|i\notin S_{3}^{k}\text{ and }i\notin S_{4}^{k}\right\}. \end{array}$$

From (11), periodic solutions must satisfy

(15) $$\begin{array}{l} {\hat{\phi }}_{1}^{1}={\hat{\phi }}_{2}^{2}=\cdots ={\hat{\phi }}_{N+1}^{N+1}\equiv {\hat{\phi }}^{\text{td}}. \end{array}$$

This means that the touchdown phase of all oscillators equals ${\hat{\phi }}^{\text{td}}$. From (14) and (15), we obtain the periodic solution by ${\hat{\phi }}^{\text{td}}$ and ${\hat{\psi }}_{i}^{k}\left(i=1,\ldots ,N\right)$ as follows:

(16) $$\begin{array}{l} {\hat{\phi }}^{\text{td}}=2\pi -m_{\text{B}}b \end{array}$$

(17) $$\begin{array}{l} {\hat{\psi }}_{i}^{k}=\left\{ \begin{array}{cc} {\hat{\psi }}_{k-1}^{k} & i=k-1\\ 2\left(1-\beta \right)\pi -\left(2m_{\text{B}}-m\right)b & i\in T_{1}^{k}\\ 2\left(1-\beta \right)\pi -\left(3m_{\text{B}}-m\right)b & i\in \left[1,k-2\right]\text{ and }i\notin T_{1}^{k}\\ {\hat{\psi }}_{k}^{k} & i=k\\ 2\beta \pi +\left(2m_{\text{B}}-m\right)b & i+1\in T_{2}^{k}\\ 2\beta \pi +\left(3m_{\text{B}}-m\right)b & \text{otherwise}, \end{array}\right. \end{array}$$

where *b* = ((1−β)*gπ*)/(κ*a*). The first row, the following two rows, and the remaining rows of the right-hand side of (17) represent the relative phases on the boundary, in the direct-wave region, and in the retrograde-wave region, respectively. The sets $T_{1,2}^{k}$ are given by

(18) $$\begin{array}{l} T_{1}^{k}=\left\{i\in \left[1,k-2\right]|2\beta \pi +\left(2m_{\text{B}}-m\right)b<{\hat{\phi }}_{i}^{k}\leq {\hat{\phi }}^{\text{td}}\right\}\\ T_{2}^{k}=\{i\in \left[k+2,N+1\right]|2\beta \pi +\left(2m_{\text{B}}-m\right)b<{\hat{\phi }}_{i}^{k}\leq {\hat{\phi }}^{\text{td}}\}. \end{array}$$

$T_{1}^{k}$ is the set of legs (Leg *i*) in the direct-wave region whose neighboring legs (Legs *i*−1 and *i*+1) do not experience phase resetting between the touchdowns of Legs *k* and *i* (i.e., $S_{3}^{k}$ in the direct-wave region). $T_{2}^{k}$ is the set of legs (Leg *i*) in the retrograde-wave region whose neighboring legs (Legs *i*−1 and *i*+1) do not experience phase resetting between the touchdowns of Legs *k* and *i* (i.e., $S_{3}^{k}$ in the retrograde-wave region). ${\hat{\psi }}_{k-1}^{k}$ is not determined uniquely but satisfies

(19) $$\begin{array}{l} 2\left(1-\beta \right)\pi -\left(3m_{\text{B}}-m\right)b<{\hat{\psi }}_{k-1}^{k}\leq 2\beta \pi . \end{array}$$

**Figure 10:**
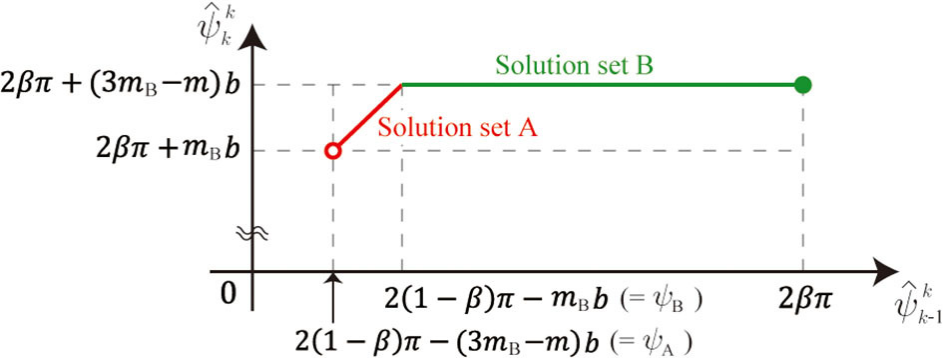
Relation between ${\hat{\psi }}_{k-1}^{k}$ and ${\hat{\psi }}_{k}^{k}$.

This non-uniqueness is because the length of Leg *i* is constant in the stance phase (i.e., *l*(ϕ_*i*_) = *L*, which does not determine ϕ_*i*_ uniquely). ${\hat{\psi }}_{k}^{k}$ is represented using ${\hat{\psi }}_{k-1}^{k}$ as

(20) $$\begin{array}{l} {\hat{\psi }}_{k}^{k}=\{\begin{array}{cc} {\hat{\psi }}_{k-1}^{k}+\left(4\beta -2\right)\pi +\left(4m_{\text{B}}-m\right)b & \psi_{\text{A}}<{\hat{\psi }}_{k-1}^{k}<\psi_{\text{B}}\\ 2\beta \pi +\left(3m_{\text{B}}-m\right)b & \psi_{\text{B}}\leq {\hat{\psi }}_{k-1}^{k}\leq 2\beta \pi \end{array} \end{array}$$

where ψ_A_ = 2(1 − β)π − (3*m*_B_ − *m*)*b* and ψ_B_ = 2(1 − β)π − *m*_B_*b*. Therefore, the relationship of ${\hat{\psi }}_{k-1}^{k}$ and ${\hat{\psi }}_{k}^{k}$ is explained by two connected segments, as shown in Figure 10. We call these segments solution sets A and B. Note that solution set A is smaller than solution set B, as shown in Figure 4, because κ ≫ 1.

**Table 2:** Classification of sets $S_{1-8}^{k}$.

	**Experience of phase resetting**	
**Set**	**Leg *i* − 1 (fore side)**	**Leg *i* + 1 (hind side)**
$S_{1}^{k}$	–	No
$S_{2}^{k}$	–	Yes
$S_{3}^{k}$	No	No
$S_{4}^{k}$	Yes	Yes
$S_{5}^{k}$	No	Yes
$S_{6}^{k}$	Yes	No
$S_{7}^{k}$	No	–
$S_{8}^{k}$	Yes	–

### 3.4. Stability of Source-Wave Gait

To discuss stability, we added perturbation $\Delta \psi_{i}^{k}\left(i=1,\ldots ,N\right)$ to the obtained periodic solutions on Σ^*k*^ and evaluated the linear stability by analytically calculating the linear map of perturbation:

(21) $$\begin{array}{l} \Delta \Psi^{k}\mapsto P_{k}\Delta \Psi^{k}, \end{array}$$

where $\Delta \Psi^{k}={\left[\Delta \psi_{1}^{k}\Delta \psi_{2}^{k}\ldots \Delta \psi_{N}^{k}\right]}^{T}$, and $P_{k}\in {\mathbb{R}}^{N\times N}$ is the Jacobian matrix of the Poincaré map. We assumed that the perturbation is too small to change the order of touchdown events and the sets *S*_*i*_.

When we write the perturbed touchdown phase of Leg *i* as ${\hat{\phi }}^{\text{td}}+\Delta \phi_{i}^{i}$ and substitute $\psi_{i}^{k}={\hat{\psi }}_{i}^{k}+\Delta \psi_{i}^{k}$ and $\phi_{i}^{i}={\hat{\phi }}^{\text{td}}+\Delta \phi_{i}^{i}$ into (14), $\Delta \phi_{i}^{i}$ (*i* = 1, …, *N* + 1) can be represented with $\Delta \psi_{i}^{k}$. The matrix *P*_*k*_ is derived by substituting $\phi_{i}^{i}={\hat{\phi }}^{\text{td}}+\Delta \phi_{i}^{i}$ into (11). The eigenvalues λ_S*k*_ of *P*_*k*_ for the source-wave gait are derived depending on solution sets A and B by

(22) $$\begin{array}{l} \lambda_{\text{S}k}=\{\begin{array}{cc} \left\{1,1/2,2/3,\ldots ,2/3\right\} & \psi_{\text{A}}<{\hat{\psi }}_{k-1}^{k}<\psi_{\text{B}}\\ \left\{1,2/3,2/3,\ldots ,2/3\right\} & \psi_{\text{B}}\leq {\hat{\psi }}_{k-1}^{k}\leq 2\beta \pi \end{array} \end{array}$$

There is only one eigenvalue of 1 for both solution sets due to the non-uniqueness. However, the other eigenvalues are <1, which means that any initial points near the solution set will converge to the solution set.

### 3.5. Analytical Solution and Stability of Direct-Wave Gait

The solution of the direct-wave gait cannot be derived by substituting *k* = *N* + 1 for those of the source-wave gaits. This is because part of the assumption regarding the phases of neighboring legs (specifically, that $\phi_{k-1}^{k}$ is in the takeoff phase, as in the middle figure of Figure 8) is not correct when *k* = *N* + 1 for the direct-wave gait. Thus, we derive the solution of the direct-wave gait from the front–rear symmetry and the solution of the retrograde-wave gait.

We denote the flow of the oscillator phase *i* (*i* = 1, …, *N* + 1) with the initial value $\left({\tilde{\phi }}_{1}^{k},{\tilde{\phi }}_{2}^{k},\ldots ,{\tilde{\phi }}_{N+1}^{k}\right)$ just before the touchdown of Leg *k* by $\Phi_{i}\left(t;{\tilde{\phi }}_{1}^{k},{\tilde{\phi }}_{2}^{k},\ldots ,{\tilde{\phi }}_{N+1}^{k}\right)$. From the front–rear symmetry of our model, the following equation is satisfied for the initial phases such that ${\tilde{\phi }}_{i}^{k}={\tilde{\phi }}_{i}^{N+2-k}$ for *i* = 1, …, *N* + 1:

(23) $$\begin{array}{l} \Phi_{i}\left(t;{\tilde{\phi }}_{1}^{k},{\tilde{\phi }}_{2}^{k},\ldots ,{\tilde{\phi }}_{N+1}^{k}\right)\\ \;=\Phi_{N+2-i}(t;{\tilde{\phi }}_{N+1}^{N+2-k},{\tilde{\phi }}_{N}^{N+2-k},\ldots ,{\tilde{\phi }}_{1}^{N+2-k}) \end{array}$$

The periodic solution of the direct-wave gait ${\hat{\psi }}_{i}^{N}\left(i=1,\ldots ,N\right)$ on Σ^*N*^, where ${\hat{\phi }}^{\text{td}}$ is the same as (16), is derived from solution set B of the retrograde-wave gait (*k* = 2 for the source-wave gait) using the symmetry condition (symmetrical solution for solution set A of the retrograde-wave gait equals solution set A of the source-wave gait with *k* = *N*). Specifically, the substitution of *k* = *N* into (23) yields $\Phi_{i}\left(t;{\tilde{\phi }}_{N+1}^{N},\ldots ,{\tilde{\phi }}_{1}^{N}\right)=\Phi_{N+2-i}\left(t;{\tilde{\phi }}_{1}^{2},\ldots ,{\tilde{\phi }}_{N+1}^{2}\right)$. When the periodic solution ${\hat{\phi }}_{j}^{N}$ (*j* = 1, …, *N* + 1) is used for the initial value ${\tilde{\phi }}_{j}^{N}$, we obtain ${\hat{\phi }}_{i}^{N}={\hat{\phi }}_{N+2-i}^{2}$ (*i* = 1, …, *N*). This yields ${\hat{\psi }}_{i}^{N}=2\pi -{\hat{\psi }}_{N+1-i}^{2}$ because ${\hat{\psi }}_{i}^{N}={\hat{\phi }}_{i+1}^{N}-{\hat{\phi }}_{i}^{N}$. By substituting solution set B of the retrograde-wave gait ${\hat{\psi }}_{N+1-i}^{2}$ (*k* = 2 in (17)), we obtain the solution of the direct-wave gait as

(24) $$\begin{array}{l} {\hat{\psi }}_{i}^{N}=\{\begin{array}{cc} {\hat{\psi }}_{N}^{N} & i=N\\ 2\left(1-\beta \right)\pi -\left(2m_{\text{B}}-m\right)b & i\in T_{1}^{N}\\ 2\left(1-\beta \right)\pi -\left(3m_{\text{B}}-m\right)b & \text{otherwise}. \end{array} \end{array}$$

where ${\hat{\psi }}_{N}^{N}$ is an arbitrary constant fulfilling

(25) $$\begin{array}{l} 2\left(1-\beta \right)\pi \leq {\hat{\psi }}_{N}^{N}\leq 2\beta \pi +m_{\text{B}}b. \end{array}$$

The linear stability of the solution can also be calculated using the symmetry condition (23). The symmetry condition gives the relation $\Delta \psi_{i}^{N}=-\Delta \psi_{N+1-i}^{2}$, which is represented as Δ**Ψ**^*N*^ = −*Q*Δ**Ψ**^2^, where the matrix *Q* is an anti-diagonal matrix with all the anti-diagonal elements of 1. By using *Q*, the Jacobian matrix of the Poincaré map of the direct-wave gait *P*_D_ is calculated as $P_{\text{D}}=QP_{2}Q^{-1}$. Thus, the eigenvalues λ_D_ of *P*_D_ are obtained by those of *P*_2_ as

(26) $$\begin{array}{l} \lambda_{\text{D}}=\left\{1,2/3,2/3,\ldots ,2/3\right\}. \end{array}$$

There is only one eigenvalue of 1 due to the non-uniqueness. However, the other eigenvalues are <1, which means that any initial points near the solution set will converge to the solution set.

**Figure 11:**
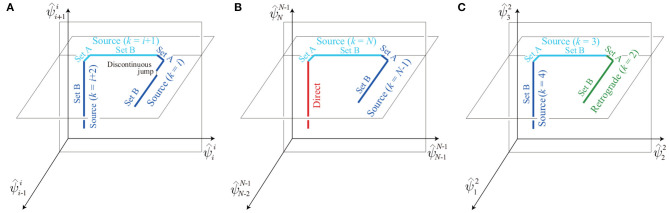
Connection of the solution sets: **(A)** Source-wave gaits (*k* = *i, i* + 1, *i* + 2) projected onto the ${\hat{\psi }}_{i-1}^{i}$-${\hat{\psi }}_{i}^{i}$-${\hat{\psi }}_{i+1}^{i}$ space; **(B)** direct- and source-wave gaits (*k* = *N*−1, *N*) projected onto the ${\hat{\psi }}_{N-2}^{N-1}$-${\hat{\psi }}_{N-1}^{N-1}$-${\hat{\psi }}_{N}^{N-1}$ space; and **(C)** retrograde- (*k* = 2) and source-wave gaits (*k* = 3, 4) projected onto the ${\hat{\psi }}_{1}^{2}$-${\hat{\psi }}_{2}^{2}$-${\hat{\psi }}_{3}^{2}$ space.

All the solution sets we derived were connected serially as a chain. Specifically, the boundary on solution set A of the source-wave gait with *k* = *i* (left side of Figure 10) and the boundary on solution set B of the source-wave gait with *k* = *i* + 1 (right side of Figure 10) are connected, as shown in Figure 11A. In addition, the boundary on solution set A of the source-wave gait with *k* = *i* + 1 and the boundary on solution set B of the source-wave gait with *k* = *i* + 2 are connected, as shown in Figure 11A. Furthermore, the boundary on solution set A of the source-wave gait with *k* = *N* and the boundary of the direct-wave gait are connected, as shown in Figure 11B. The boundary on solution set B of the source-wave gait with *k* = 3 and the boundary on solution set A of the source-wave gait with *k* = 2 (retrograde-wave gait) are connected, as shown in Figure 11C. Thus, the obtained solution sets consist of many connected segments constrained in different planes, and the boundaries of the whole solution set correspond to the direct- and retrograde-wave gaits. Note that discontinuous jumps may exist on the analytical solution sets, as highlighted in Figure 11A. The jumps occur when the elements of the sets *T*_1_ and *T*_2_ change, that is, when the order of touchdown events of the neighboring legs changes. For example, when Leg *s* becomes an element of $T_{1}^{k}$, that is, when the order of touchdown events of the neighboring legs changes from Legs *s*+1, *s*, and *s*−1 to Legs *s*, *s*−1, and *s*+1, the relative phase ${\hat{\psi }}_{s}^{k}$ jumps from 2(1−β)π−(3*m*_B_−*m*)*b* to 2(1−β)π−(2*m*_B_−*m*)*b*, as shown in (17).

### 3.6. Comparison With Simulation Results

To validate the analytical results, we compared the obtained solutions for *N* = 3 with the simulation results on Σ^1^ in Figure 4, where the analytical solutions were derived using the same parameters of the simulation. In both the simulation and analytical results, source- and retrograde-wave gaits show two segments as solution sets A and B (although some discontinuous jumps appear). The segments of three gaits are serially connected, and their edges correspond to the direct- and retrograde-wave gaits. These characteristics were identical between the analytical and simulation results. Furthermore, the maximum distance between the solution sets of the simulation and analytical results was only 0.11 (1.8% of 2π). The errors mainly came from discontinuous jumps in the analytical solution, which are not shown in the simulation, because the first-order lag system in (5) smoothed the jumps.

### 3.7. Geometrical Features of Solutions

From the analytical solution (17), we found that the swing movements of the source-wave gait are isotropic waves and propagate forward from Leg *k* − 1 (direct-wave region) and backward from Leg *k* (retrograde-wave region) with constant speeds. This gait is similar to that observed in millipedes (Tamura et al., 2016). When the interval between the legs is η, the wavelength ξ and velocity *v* of the swing movement are derived by

(27) $$\begin{array}{l} \xi =\frac{\frac{1}{1-\beta }-\frac{m_{\text{B}}g}{2\kappa a}}{1-\frac{\left(3m_{\text{B}}-m\right)g}{2\kappa a}}\eta , \end{array}$$

(28) $$\begin{array}{l} v=\frac{\xi \omega }{2\pi }. \end{array}$$

As the duty factor β increases, ξ and *v* increase.

When *k* = 2 and ${\hat{\psi }}_{1}\approx 2\beta \pi$, the swing movements of the solution propagate from anterior to posterior with the wavelength from (27) and velocity from (28). This is similar to the retrograde-wave gait of centipedes (Full, 1997; Kuroda et al., 2014). In contrast, when ${\hat{\psi }}_{N}\approx 2\left(1-\beta \right)\pi$ in (24), the solution, whose front and rear parts are reversed from the solution of the retrograde-wave gait, is similar to the direct-wave gait of millipedes (Full, 1997; Kuroda et al., 2014). Our model has solutions corresponding to direct-, retrograde-, and source-wave gaits for *N* ≥ 3.

## 4. Discussion

### 4.1. Body Elasticity and Local Sensory Feedback Generate Interlimb Coordination

We assume that animal gaits can be represented as attractors of dynamic systems, as in Schöner et al. (1990), and that the essential structure of the dynamic system can be extracted by using a simple model (Full and Koditschek, 1999). Accumulating an understanding of such simple models allows us to understand actual complex phenomena. In the present study, we focused on the embodied sensorimotor interaction to generate multi-legged locomotion in a decentralized manner. We showed that the local sensory feedback and phase resetting generates the direct-, retrograde-, and source-wave gaits observed in multi-legged animals using a simple model to extract the essential features. We found that body elasticity is a key to generating the interlimb coordination. Specifically, the body natural frequency must be larger than the gait frequency ($\sqrt{\kappa /m_{i}}\gg \omega$). The analytical representation showed the existence of these gaits for *N* ≥ 3, and revealed the parameter domain as follows: the boundary condition of the mass, *m*/2 < *m*_B_ < *m*, and the amplitude of the swing leg movement, *mg*/κ < *a* < *L*. These findings improve our understanding of the mechanism of interlimb coordination in multi-legged locomotion.

So far, many studies have investigated the effects of sensory feedback on interlimb coordination. Owaki et al. (2012), Fukuoka et al. (2015), and Owaki and Ishiguro (2017) showed that quadruped robots, whose legs are controlled by distributed oscillators with load sensory feedback, generate walking, trotting, and galloping gaits depending on the speed. Tamura et al. (2016), Kano et al. (2017), and Yasui et al. (2017) also showed that load sensory feedback generates a millipede-like direct-wave gait. Our previous studies showed that phase resetting induces gait transitions in quadruped locomotion (Aoi et al., 2011, 2013) and hexapod locomotion (Fujiki et al., 2013; Ambe et al., 2015, 2018). In the present study, we also demonstrated that phase resetting generates retrograde-, source-, and direct-wave gaits in multi-legged locomotion. These findings are due to the embodied sensorimotor interaction, which is critical for the interlimb coordination regardless of the number of legs. Our analytical description using a simple model is helpful for clarifying the functional roles of the embodied sensorimotor interaction in interlimb coordination.

### 4.2. Relation With Intersegmental Coordination in Other Organisms

Other organisms also show intersegmental coordination in their locomotion, which appears as waves. For example, insect larvae and worms exhibit direct waves while crawling (Trimmer and Issberner, 2007; Paoletti and Mahadevan, 2014). Lampreys, leeches, and roundworms (*C. Elegans*) show body undulations that propagate backward during swimming. Motile cilia show metachronal waves. We discuss the relationship between our results and such intersegmental coordination by focusing on local sensory feedback.

Paoletti and Mahadevan (2014) developed an earthworm crawling model using mass points connected by spring dampers in an asymmetric friction environment. The model is controlled by neuro-muscular dynamics with the local sensory feedback, which contracts the segment muscles based on a stretch threshold and generates metachronal waves. Umedachi et al. (2016) proposed a similar model to reproduce the direct wave crawling motion of larvae in a decentralized manner. Each segment repeats stretching and contraction with distributed CPGs with local sensory feedback of the friction and velocity information for the segment. These results are similar to ours in the sense that metachronal waves are generated by local sensory feedback. However, their coordination is generated by asymmetric environmental friction, whereas ours is by different foot-contact timings.

The undulation patterns of lampreys and leeches as they swim are mainly generated by internal coupling of CPGs (Cohen et al., 1992; Grillner et al., 1995; KristanJr. et al., 2005). However, intersegmental coordination in leeches is not disrupted much even if the ganglion is cut (Yu et al., 1999), which suggests the importance of sensory feedback for segmental coordination. In addition, there is no clear evidence for the presence of CPGs in *C. Elegans*, and it has been suggested that proprioceptive receptors play a significant role in coordination (Wen et al., 2012). Boyle et al. (2012) modeled *C. Elegans* as a series of simple segments connected by elastic elements. They reproduced body undulations by neuro-muscular dynamics without CPGs. The dynamics of each segment is affected by the sensory feedback, which integrates several posterior segment stretches. When the feedback has only local interaction, such as stretch of the segment, no coordinated wave appears in a less viscous environment, such as water, and it appears only in a highly viscous fluid environment. That is, the fluid viscosity is responsible for generating the coordinated wave.

Motile cilia of organisms generate metachronal waves in a decentralized manner due to local interaction between the environmental fluid and the cilia (Elgeti and Gompper, 2013). Each cilium is controlled by simple switching inputs of power stroke and return stroke. These inputs switch when the cilium achieves a certain curvature; that is, it receives local sensory feedback about the curvature. Although each cilium moves independently, the metachronal wave for cilia motion appears through local interaction by the flow of the neighboring fluid.

An analytical description would help to understand these intersegmental coordination mechanisms. Thus, in the future, we would like to develop simple physical models for intersegmental coordination.

### 4.3. Physical Explanation of Countless Solutions

Our model has countless solutions as obtained by the serially connected set of the retrograde-, source-, and direct-wave gaits (Figure 11). This non-uniqueness is because our model has a conservative quantity as explained below.

We define *E*^*j*^ on the Poincaré section Σ^*j*^ (*j* ∈ [1, *N* + 1]) by

(29) $$\begin{array}{l} E^{j}=\overset{N}{\underset{i=1}{\sum }}\psi_{i}^{j}. \end{array}$$

From (11), the Poincaré map of *E*^*j*^ is represented as

(30) $$\begin{array}{l} E^{j}\mapsto E^{j}+\phi_{1}^{1}-\phi_{N+1}^{N+1}. \end{array}$$

The relation $\phi_{1}^{1}=\phi_{N+1}^{N+1}={\hat{\phi }}^{\text{td}}$ holds if the neighboring legs (Legs 2 and *N*) are in the stance phases ($\phi_{2}^{1}\in \left[0,2\beta \pi \right]$ and $\phi_{N}^{N+1}\in \left[0,2\beta \pi \right]$) when each leg (Legs 1 and *N* + 1) touches the ground (this relation is also satisfied for the solution sets). In this case, (30) becomes *E*^*j*^ ↦ *E*^*j*^, where *E*^*j*^ is a conservative quantity, which produces countless solutions.

To explain the physical meaning of this conservative quantity, we represent $\phi_{1}^{1}$ and $\phi_{N+1}^{N+1}$ using (3) and (12) when $\phi_{2}^{1}\in \left[0,2\beta \pi \right]$ and $\phi_{N}^{N+1}\in \left[0,2\beta \pi \right]$.

(31) $$\begin{array}{l} \phi_{1}^{1}=2\pi -\frac{\left(1-\beta \right)m_{1}g\pi }{\kappa a}, \end{array}$$

(32) $$\begin{array}{l} \phi_{N+1}^{N+1}=2\pi -\frac{\left(1-\beta \right)m_{N+1}g\pi }{\kappa a}. \end{array}$$

These equations show that $\phi_{1}^{1}=\phi_{N+1}^{N+1}$ is satisfied only when *m*_1_ = *m*_*N* + 1_. Thus, this conservative quantity is derived from the symmetry between the front and tail masses (*m*_1_ = *m*_*N* + 1_ = *m*_B_). Strictly speaking, the symmetry of other parameters, such as κ and *a*, is also required for the conservative quantity.

Although our model generates multiple gaits because of the symmetry, animals prefer specific gaits. For example, centipedes prefer the retrograde-wave gait (Full, 1997; Kuroda et al., 2014). In contrast, millipedes prefer the direct-wave gait to move forward (Full, 1997; Kuroda et al., 2014) and use the source-wave gait when the body axis is bent like a U shape (Tamura et al., 2016). Our model does not explain these preferences. However, when the symmetry is broken, our model has no conservative quantity and generates a specific gait depending on the parameters. We would like to investigate the gait preference by incorporating asymmetries into our model in future studies.

### 4.4. Limitation of This Study

This study demonstrated that phase resetting generates direct-, retrograde-, and source-wave gaits in an elastic body whose natural frequency is larger than the gait frequency. However, our model did not replicate the details of animal gaits. For example, our model did not explain the gait preference of animals, as described above. While centipedes and millipedes use different contralateral interlimb coordination (i.e., left–right antiphase and in-phase movements, respectively), our model did not consider this behavior. Furthermore, although the swinging of multiple legs propagates simultaneously in millipedes and centipedes (Kuroda et al., 2014), only one leg swing is propagated in our model. These limitations and discrepancies come from the simple way in which our model extracts the essence of multi-leg motion, which was the focus in the present study. However, our results give clues to overcome these limitations and discrepancies. For example, the gait preference may be explained by introducing an asymmetry into our model, such as different masses between the head and tail, to eliminate the conservative quantity in our model. Better understanding of a simple model will provide insights for the design of a more complicated model. The extension of our model is one of our essential future tasks.

### 4.5. Application to Legged Robots

Multi-legged robots have been developed to extract the essence of dexterous traveling ability. On one hand, the flexible body axis contributes to rapid movement or ability to traverse rough terrain. In previous work (Aoi et al., 2016), we showed that high body-axis flexibility induces body undulations through Hopf bifurcation, which contributes to rapid turning. Hoffman and Wood (2011) showed that a passive undulatory gait increases the locomotion speed. Koh et al. (2010) and Kinugasa et al. (2017) demonstrated that multi-legged robots with a flexible body axis show high mobility in uneven terrain.

On the other hand, contacting the ground with many legs is useful for avoiding stumbling in various environments. Inagaki et al. (2010) proposed a distributed control method in which the legs follow the contact points of anterior legs, which allowed a robot to walk in various environments as long as the front legs choose solid footholds. Hayakawa et al. (2020) proposed a gait generation strategy to ensure static stability for single-legged modular robots to create a cluster with various leg configurations. Kano et al. (2017) showed the adaptability when part of the terrain is removed.

For static gaits, sensory feedback is useful for generating interlimb coordination, as in the present study, and to gain better adaptability to the environment (Kano et al., 2017; Yasui et al., 2017). To design a method for controlling multi-legged robots with sensory feedback, it is important to understand how and when the sensory feedback affects walking motion. The present study focused on phase resetting at foot contact as the sensory feedback and clarified the effects by using the analytical description of a simple model. We showed that phase resetting contributes to generating the coordinated gaits in a decentralized manner via embodied sensorimotor interaction when the model has an elastic body axis whose natural frequency is larger than the gait frequency. We also analytically derived the range of physical parameters where our analysis is valid. This helps when designing a controller to generate various gaits for multi-legged robots. In particular, when we design the leg movement using oscillator phases, as in Aoi et al. (2017) and Ambe et al. (2018), phase resetting will generate coordinated gaits regardless of the number of legs. We would like to investigate it through robot experiments in future studies.

## 5. Conclusion

This study used a simple model to analytically reveal that local sensory feedback, phase resetting, generates the direct-, retrograde-, and source-wave gaits observed in multi-legged animals in a decentralized manner via the embodied sensorimotor interaction. The model comprises massless legs and mass points connected by vertical springs to imitate a flexible body. The phase oscillators control the vertical movements of the legs independently, and the phases are only affected by phase resetting upon foot contact. The dynamic simulations show that countless periodic solutions of the three gaits emerge depending on the initial phase. Furthermore, these analytical solutions were derived under some assumptions deduced from the simulation results. They showed that all three gaits exist regardless the number of legs and revealed the parameter domain. The reason for the coexistence of the three gaits is explained by a conservative quantity due to the front–rear symmetry of our model. Because our model is limited to specific situations, such as the front–rear symmetry, permitting only vertical movements while ignoring the contralateral interlimb coordination, and propagation of only one swing leg within one wavelength, we need to incorporate more realistic situations into our model in the future.

## Data Availability Statement

The original contributions presented in the study are included in the article/Supplementary Material, further inquiries can be directed to the corresponding author/s.

## Author Contributions

YA conceived of the study, designed the study, carried out the simulation and theoretical analysis, and drafted the manuscript. SA helped conceiving and designing of the study, improved the theoretical analysis, and critically revised the manuscript. KT helped conceiving and designing of the study and critically revised the manuscript. FM helped conceiving and designing of the study, coordinated the study, and critically revised the manuscript. All authors gave final approval for publication and agree to be held accountable for the work performed therein.

## Conflict of Interest

The authors declare that the research was conducted in the absence of any commercial or financial relationships that could be construed as a potential conflict of interest.

## Publisher's Note

All claims expressed in this article are solely those of the authors and do not necessarily represent those of their affiliated organizations, or those of the publisher, the editors and the reviewers. Any product that may be evaluated in this article, or claim that may be made by its manufacturer, is not guaranteed or endorsed by the publisher.
